# Health and disability – a multi-group latent class analysis of the World Health Organization Disability Assessment Schedule 2.0 among those with mental and physical health conditions

**DOI:** 10.1186/s12955-024-02273-8

**Published:** 2024-07-27

**Authors:** Vanessa Seet, Edimansyah Abdin, Anitha Jeyagurunathan, Tan Sing Chik, Lum Joon Kit, Lee Eng Sing, Swapna Verma, Wei Ker-Chiah, Pamela Ng, Mythily Subramaniam

**Affiliations:** 1 Research Division, Institute of Mental Health, Buangkok Green Medical Park, 10 Buangkok View, Singapore, 539747 Singapore; 2 Clinical Research Unit, National Healthcare Group Polyclinics, Singapore, Singapore; 3 Department of Psychosis, Institute of Mental Health, Singapore, Singapore; 4 West Region, Institute of Mental Health, Singapore, Singapore; 5 East Region, Institute of Mental Health, Singapore, Singapore; 6 Saw Swee Hock School of Public Health, National University of Singapore, Singapore, Singapore

**Keywords:** WHODAS, Disability, Chronic conditions, Assessment, Mental illness, Psychiatry

## Abstract

**Background:**

This study aims to identify disability classes among people with schizophrenia spectrum disorder, depression, anxiety or diabetes via the WHODAS 2.0; investigate the invariance of disability patterns among the four diagnostic groups; and examine associations between disability classes and sociodemographic variables.

**Methods:**

Patients seeking treatment for schizophrenia spectrum disorder, depression, anxiety or diabetes (n=1076) were recruited. Latent class analysis was used to identify disability classes based on WHODAS 2.0 responses. Measurement invariance was tested using multi-group latent class analysis. Associations between classes and sociodemographic variables were tested via multinomial logistic regression.

**Results:**

A five-class solution was identified; examination of model invariance showed that the partially constrained five-class model was most appropriate, suggesting that class structure was consistent while class membership differed across diagnostic groups. Finally, significant associations were found between class membership and ethnicity, education level, and employment status.

**Conclusions:**

The results show the feasibility of using the WHODAS 2.0 to identify and compare different disability classes among people with mental or physical conditions and their sociodemographic correlates. Establishing a typology of different disability profiles will help guide research and treatment plans that tackle not just clinical but also functional aspects of living with either a chronic psychiatric or physical condition.

## Background

Disability is prevalent in a large proportion of the global population, and its consequences are far-reaching and varied, with a significant impact at the individual, country, and global level. In the World Report on Disability, estimates derived from the 2004 Global Burden of Disease study pointed to 15.3% of the global population experiencing “moderate or severe disability” in their functioning (roughly 978 million people), while about 2.9% of the population (about 185 million people), experienced “severe disability” [1], cementing its status as a global health issue. At the individual level, the extra costs incurred for an individual to overcome their disabilities and reach a non-disabled standard of living range from 9% to 69% more than their non-disabled counterparts when matched for income level [2, 3]. At the country level, these extra costs are compounded by the direct (for example, disability benefits) and indirect costs (for example, labor productivity loss) of disability [4]. In order to mitigate the economic and health burdens caused by disability, research into what kinds of disability are experienced by individuals, and how best to address them, is imperative.

According to the International Classification of Functioning, Disability and Health, disability refers to difficulties in bodily functions, engaging in activities, or participating in any area of life [5]. Separate from disease severity, it is a useful indicator of health-related quality of life (QOL), a multidimensional domain that includes social and physical functioning [6]. In addition, disability is a crucial component for the evaluation of disease burden and in turn the effectiveness of health interventions [7]. It results from an interplay of health conditions, either physical or mental, and environmental factors, with varying ramifications depending on what type of disability is manifested [8, 9]. In turn, the individual’s functioning and QOL is compromised [1]. While different diseases and health conditions can vary in symptomology, disability is something that is universally experienced by almost everyone at some point in their lives. Hence, in the face of significant heterogeneity in the clinical presentations of diseases, disability is an important measure of the impact of disease, and allows for comparison across different health conditions as well as evaluating treatment efficacy in terms of functional, everyday recovery outcomes beyond reducing symptoms [10]. Given its usefulness as an indicator of health and functioning, and the multi-faceted nature of its implications, it is essential to have a robust measure of disability as a first step in assessing the various difficulties that individuals may face in their everyday lives, the resulting impact on their QOL, and the effectiveness with which they can be addressed by health interventions. It is important to distinguish between different patterns of disability people face in addition to assessing severity levels, in order to ensure that intervention programs are matched to appropriately target the individual’s specific disability, and in turn optimize outcomes.

The World Health Organization Disability Assessment Schedule 2.0 (WHODAS 2.0) is one such measure that reconciles different definitions of disability into a single scale. It takes into account the various aspects of disability - cognition, mobility, personal care, social communication and interaction, daily life activities, and societal engagement [7]. It was developed as a cross-culturally adaptable scale of health status and QOL, specifically to measure functioning and disability in the various domains of everyday life [7]. The validity and reliability of the 12-item WHODAS have been demonstrated across different settings, from general community samples [11, 12] to groups with physical [13] and mental [14, 15] conditions.

While the psychometric properties of the WHODAS 2.0 have been extensively researched and demonstrated, there is a dearth in the literature on its ability as a measure to distinguish between different disability patterns that people with different health conditions experience. To the best of our knowledge, only two studies have examined disability and severity patterns using the 12-item WHODAS 2.0 [16, 17]. However, neither examined the potential heterogeneity of such patterns between respondent groups with different diagnostic characteristics – for example, people with mental health conditions versus people with chronic physical conditions. As identifying disability patterns and their heterogeneity is paramount for better targeted interventions, we aim to bridge this literature gap by a) identifying classes of disability via WHODAS 2.0 responses; b) examining if response patterns yielding these disability classes differed between people with different chronic conditions; and c) examining sociodemographic correlates of disability classes among patients.

## Materials and methods

### Participants

The data analyzed were collected as part of a larger study on the validation of the 12-item WHODAS 2.0 among those with mental and physical illness in Singapore. Participants were recruited from the Institute of Mental Health, Singapore (IMH), Community Wellness Clinics (CWCs) of IMH, and a primary care clinic i.e., the National Healthcare Group Polyclinic (NHGP) from 2019 to 2022. Potential participants were recruited for the study if they met the following inclusion criteria: a) being 21 years of age and above, b) seeking treatment for a primary diagnosis of a schizophrenia spectrum disorder or other psychotic disorders, a depressive or anxiety disorder, or diabetes, and c) having Singaporean citizenship or Permanent Residency in Singapore. Participants with a psychiatric disorder were recruited from IMH and the CWCs, while participants with diabetes were recruited from NHGP. Upon giving their written informed consent, participants completed a series of written questionnaires and interviewer-administered scales. Ethics approval was obtained from the Institutional Research Review Committee and the National Healthcare Group Domain Specific Review Board (Ref: 2018/00772), and all study procedures were carried out in accordance with the relevant guidelines and regulations.

### World Health Organization Disability Schedule 2.0 – 12 items (WHODAS 2.0)

The WHODAS 2.0 uses a 5-item Likert-type response scale, with responses ranging from “none” or no difficulty to “extreme or cannot do”, and higher scores indicating greater disability and impairment. It has previously been validated in Singapore, with a high internal consistency of Cronbach’s α = 0.92 [12]. For ease of latent class estimation, responses were dichotomised into 0 – indicating “no to mild difficulty”, and 1 – indicating “moderate to extreme difficulty” [16].

### Sociodemographic information

Participants were asked about their sociodemographic information including age, gender, ethnicity, education level, marital status, and employment status. Of the original six levels of education, graduation from pre-university or Junior College level, having a polytechnic diploma, or graduation from a vocational institute or the Institute of Technical Education were collapsed into tertiary education, yielding four levels – primary, secondary, tertiary and university education. Of the original five categories of marital status, those who had never been married, separated, divorced or widowed were collapsed into a single category – currently unmarried – versus those who were currently married. Finally, the original nine categories of employment status were collapsed into three – those who were currently working, currently not working, and economically inactive (i.e., students and homemakers).

### Statistical analyses

To address the first study aim, latent classes were first derived from WHODAS item response probabilities in the overall sample. Latent class analysis (LCA) is a cross-sectional mixture modeling technique used to identify subgroups, or classes, of individuals in a given population through their behavioral or response patterns [18, 19]. One- to six-class models were fitted to the data and their fit assessed. Akaike Information Criteria (AIC), Bayesian Information Criteria (BIC) and sample size-adjusted BIC values were computed to evaluate model fit – smaller values indicate better model fit. Vuong-Lo-Mendell-Rubin and parametric bootstrapped likelihood ratio tests (LRTs) were computed to determine the significance improvement in fit values for the class model being tested (*k*-class) versus the class model that is one class smaller (*k*-1). Significant LRT values (i.e., *p* < 0.05) indicate that the current class solution is better than the previous k-1 class solution. The best fitting model was then selected after taking into account the fit values holistically, the principle of parsimony, and model interpretability and stability.

To address the second study aim, we employed the multi-group latent class analytical approach to examine measurement invariance of the latent class model and potential differences in latent class characteristics among the four different diagnostic groups [20]. To this end, the best-fitting model was derived within a K-class solution after comparing the following nested and full models – a) a fully constrained model in which measurement invariance was assumed and the response probabilities and class sizes were constrained; b) a semi-constrained model in which response probabilities were fixed and class sizes free to vary; and c) a fully unconstrained model, in which both response probabilities and class sizes were free to vary (the equivalent of fitting four separate models for the different diagnostic groups) [21]. Selection of the optimal model was based on comparing the constrained (nested) models’ fit against the corresponding unconstrained (fuller) models.

Finally, multinomial logistic regression was used to investigate associations between sociodemographic variables and the different classes of disability, based on posterior class membership probabilities. All analyses were conducted with Mplus version 8.2 [22].

## Results

### Overall sample

Overall, 1076 responses were analyzed. Table 1 presents the sociodemographic characteristics of all respondents and class sizes, overall and stratified by diagnostic group. About half of all respondents (48.9%) had a co-morbid chronic physical condition in addition to the primary condition they were seeking treatment for (schizophrenia spectrum disorder, depression, anxiety, or diabetes). This number is largely inflated by those seeking treatment for diabetes, with about 95% having a co-morbid physical condition. About a quarter of all respondents (27%) had a co-morbid psychiatric condition. Majority of the respondents were of Chinese ethnicity (67.1%), had a tertiary education (44.2%), were currently married (56.4%), and currently employed (60.5%)

**Table 1:** Sample characteristics

	Overall (*n*=1076)		Schizophrenia (*n*=279)		Depression (*n*=278)		Anxiety (*n*=241)		Diabetes (*n*=278)	
	mean / n	SD / %	mean / n	SD / %	mean / n	SD / %	mean / n	SD / %	mean / n	SD / %
**Age**	40.88	14.70	37.44	11.29	34.22	12.78	36.57	14.64	54.73	9.85
**Gender**										
Male	560	52	136	48.7	141	50.7	109	45.2	174	62.6
Female	516	48	143	51.3	137	49.3	132	54.8	104	37.4
**Ethnicity**										
Chinese	722	67.1	197	70.6	187	67.3	181	75.1	157	56.5
Malay	149	13.8	41	14.7	46	16.5	25	10.4	37	13.3
Indian	144	13.4	30	10.8	24	8.6	20	8.3	70	25.2
Other	61	5.7	11	3.9	21	7.6	15	6.2	14	5
**Education**										
Primary	47	4.4	16	5.7	13	4.7	2	0.8	16	5.8
Secondary	260	24.2	77	27.6	40	14.4	36	14.9	107	38.5
Tertiary	476	44.2	135	48.4	129	46.4	122	50.6	90	32.4
University	291	27	51	18.3	94	33.8	81	33.6	65	23.4
**Marital status**										
Married	607	56.4	225	80.6	185	66.5	159	66	38	13.7
Unmarried	469	43.6	54	19.4	93	33.5	82	34	240	86.3
**Employment status**										
Employed	651	60.5	135	48.4	171	61.5	143	59.3	202	72.7
Unemployed	261	24.3	120	43	64	23	55	22.8	22	7.9
Economically inactive	160	14.9	24	8.6	43	15.5	43	17.8	50	18
**Presence of co-morbidity**										
Physical co-morbidity	526	48.9	114	40.9	89	32	59	24.5	264	95
Psychiatric co-morbidity	290	27	73	26.2	103	37.1	108	44.8	6	2.2
**Class**										
Class 1 - Extensive difficulty	108	10	34	12.2	36	13	25	10.4	13	4.7
Class 2 - Cognitive and social difficulty	147	13.7	32	11.5	64	23	45	18.7	6	2.2
Class 3 - Physical difficulty	140	13	58	20.8	16	5.8	15	6.2	51	18.4
Class 4 - Emotional difficulty	255	23.7	37	13.3	114	41	98	40.7	6	2.2
Class 5 - No difficulty	426	39.6	118	42.3	48	17.3	58	24.1	202	72.7

### Latent classes in overall sample

The model fit statistics for the 1- to 6-class models using the full sample are presented in Table 2. Across the models, while the 6-class solution had the lowest AIC, BIC and sample size-adjusted BIC values, the lack of significance of the Lo-Mendell Rubin LRT indicated that it was not significantly better than the 5-class solution. Hence, the 5-class solution (AIC = 10599.23, BIC = 10918.01, sample size-adjusted BIC = 10714.74, LMR *p* < 0.05, parametric bootstrapped *p* < 0.001) was selected as it was more parsimonious and had better class separation (entropy = 0.85).

**Table 2:** Latent class models of combined sample: model fit

Model	AIC	BIC	Sample size-adjusted BIC	Lo–Mendell‐Rubin LRT *p*‐value	Parametric bootstraped LRT *p*-value	Entropy
1 class	14375.62	14435.39	14397.28	-		
2 class	11339.44	11463.96	11384.56	<0.001	<0.001	0.884
3 class	10848.41	11037.69	10916.99	<0.001	<0.001	0.852
4 class	10713.5	10967.53	10805.55	0.003	<0.001	0.83
**5 class**	**10599.23**	**10918.01**	**10714.74**	**0.0265**	**<0.001**	**0.853**
6 class	10509.29	10892.82	10648.26	0.3139	<0.001	0.835

**Fig. 1:**
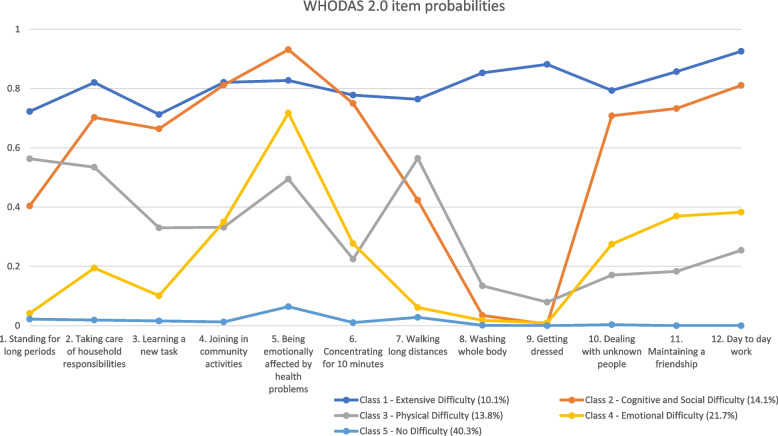
WHODAS 2.0 item response probabilities for the 5-class model

Figure 1 depicts the plotted conditional WHODAS 2.0 item probabilities associated with each class in the overall sample. Class 1, the smallest class at 10.1% of the overall sample, is characterized by high probabilities of endorsing difficulty in all items. Hence, this class depicts “extensive difficulty”. Class 2 (14.1% of sample), “cognitive and social difficulty”, is characterized by high probabilities of difficulty in learning a new task, joining in community activities, concentration, dealing with strangers, maintaining friendships, and day-to-day work. Class 3 (13.8%), “physical difficulty”, is characterized by moderate probabilities of difficulty in standing for long periods, walking long distances, and taking care of household responsibilities. Class 4 (21.7%), “emotional difficulty”, is characterized by a high probability of being emotionally affected by health problems only. Finally, Class 5, “no difficulty”, is characterized by low probabilities of difficulty in all items. The majority of respondents belonged to this class (40.3%).

### Multiple-group LCA model

After determining the five-class latent class model had optimal fit, model invariance was then examined using multiple-group LCA. Table 3 presents the fit statistics and parameters of the fully constrained, partially constrained and fully unconstrained 5-class models. The fully unconstrained model had the lowest AIC value, while the partially constrained model had the lowest BIC and sample size-adjusted BIC values. Because the BIC and sample size-adjusted BIC perform better at true model identification [23, 24], and the reduction in AIC from the partially constrained model to the fully unconstrained model is small given the corresponding increase in number of free parameters, the partially constrained model was selected as the most appropriate model. Hence, the results indicate that it is reasonable to assume measurement invariance of the classes across the diagnostic groups, while class membership differs across the groups.

**Table 3:** Fit statistics of 5-class models (invariance testing)

	Number of parameters	AIC	BIC	Sample size-adjusted BIC	Entropy
Fully constrained ^a^	67	13584.55	13918.28	13705.47	0.921
Partially constrained ^b^	**79**	**13393.47**	**13786.97**	**13536.05**	**0.901**
Fully unconstrained ^c^	259	13358.48	14648.57	13825.93	0.932

^a^item response probabilities and class sizes were constrained

^b^item response probabilities were constrained, and class sizes free to vary

^c^item response probabilities and class sizes were both free to vary

### Class membership associations

Table 4 depicts the results of the regression model, controlled for diagnostic group, physical and psychiatric co-morbidity, and with Class Five (“no difficulty”) as the reference class. With regards to ethnicity, compared to their Chinese counterparts, those who were Malay (OR = 3.471, 95% CI = 1.75 – 6.89, *p* < 0.05) or Indian (OR = 3.69, 95% CI = 1.91 – 7.15, *p* < 0.05) were more likely to belong to the extensive difficulty class. Those of Indian ethnicity were also more likely to belong to the physical difficulty class (OR = 2.99, 95% CI = 1.73 – 5.17, *p* < 0.05). Compared to people with a primary education, those who had a tertiary or university education were less likely to be in Classes One or Two, while those with a university education were less likely to be in Class Four. Finally, being unemployed was associated with higher odds of being in Class One as opposed to Class Five (OR = 2.79, 95% CI = 1.59 – 4.89, *p* < 0.05).

**Table 4:** Multinomial logistic regression results

	OR	SE	95% CIs			p
**Class 1 - Extensive Difficulty**						
Age	0.976	0.012	0.953	-	0.999	0.041
Gender						
Male (ref)						
Female	0.921	0.221	0.576	-	1.474	0.722
Ethnicity						
Chinese (ref)						
Malay	3.471	1.213	1.75	-	6.886	0.042
Indian	3.69	1.244	1.905	-	7.146	0.031
Other	5.544	2.712	2.126	-	14.46	0.094
Education						
Primary (ref)						
Secondary	0.772	0.426	0.262	-	2.276	0.592
Tertiary	0.304	0.168	0.103	-	0.897	<0.001
University	0.148	0.088	0.046	-	0.476	<0.001
Marital status						
Married (ref)						
Unmarried	0.765	0.246	0.407	-	1.436	0.339
Employment status						
Employed (ref)						
Unemployed	2.786	0.8	1.586	-	4.892	0.026
Economically inactive	0.966	0.361	0.465	-	2.008	0.924
**Class 2 - Cognitive and Social Difficulty**						
Age	0.93	0.013	0.905	-	0.956	<0.001
Gender						
Male (ref)						
Female	0.868	0.195	0.559	-	1.349	0.499
Ethnicity						
Chinese (ref)						
Malay	1.716	0.575	0.889	-	3.311	0.213
Indian	1.345	0.521	0.629	-	2.875	0.508
Other	3.156	1.49	1.251	-	7.963	0.148
Education						
Primary (ref)						
Secondary	0.849	0.498	0.269	-	2.683	0.762
Tertiary	0.305	0.176	0.099	-	0.942	<0.001
University	0.172	0.104	0.052	-	0.565	<0.001
Marital status						
Married (ref)						
Unmarried	1.039	0.356	0.531	-	2.034	0.913
Employment status						
Employed (ref)						
Unemployed	1.674	0.485	0.949	-	2.954	0.165
Economically inactive	0.821	0.278	0.422	-	1.595	0.52
**Class 3 - Physical Difficulty**						
Age	1.003	0.011	0.982	-	1.024	0.792
Gender						
Male (ref)						
Female	1.167	0.245	0.773	-	1.76	0.496
Ethnicity						
Chinese (ref)						
Malay	1.997	0.652	1.053	-	3.787	0.126
Indian	2.989	0.835	1.729	-	5.167	0.017
Other	3.067	1.484	1.188	-	7.919	0.164
Education						
Primary (ref)						
Secondary	1.919	1.081	0.636	-	5.787	0.395
Tertiary	1.196	0.68	0.392	-	3.645	0.773
University	0.732	0.441	0.225	-	2.386	0.544
Marital status						
Married (ref)						
Unmarried	0.702	0.2	0.401	-	1.228	0.137
Employment status						
Employed (ref)						
Unemployed	1.383	0.372	0.816	-	2.342	0.304
Economically inactive	0.946	0.291	0.517	-	1.73	0.852
**Class 4 - Emotional Difficulty**						
Age	0.942	0.01	0.923	-	0.962	<0.001
Gender						
Male (ref)						
Female	1.097	0.211	0.752	-	1.599	0.646
Ethnicity						
Chinese (ref)						
Malay	1.438	0.451	0.778	-	2.658	0.332
Indian	1.309	0.454	0.663	-	2.584	0.496
Other	1.944	0.841	0.833	-	4.537	0.262
Education						
Primary (ref)						
Secondary	0.531	0.304	0.173	-	1.63	0.123
Tertiary	0.382	0.213	0.128	-	1.139	0.004
University	0.308	0.175	0.101	-	0.935	<0.001
Marital status						
Married (ref)						
Unmarried	1.063	0.291	0.622	-	1.818	0.828
Employment status						
Employed (ref)						
Unemployed	1.687	0.411	1.046	-	2.721	0.095
Economically inactive	0.732	0.213	0.414	-	1.294	0.208

All models controlled for diagnostic group and co-morbidity of chronic physical and mental health conditions

## Discussion

Our study examined the different disability profiles of people with a chronic physical or mental health condition, specifically those with a schizophrenia spectrum disorder, depressive, or anxiety disorder, and diabetes. While previous studies have investigated latent class profiles of disability among people with different conditions, we expanded on these studies by employing multiple-group latent class analysis to examine if disability patterns as captured by the WHODAS 2.0 can be profiled similarly across people with mental and physical health conditions. Via latent class analysis, five classes were derived from the response patterns of the study sample, and the subsequent 5-class model was found to be consistent across the four diagnostic groups.

Contrary to previous studies which found four-class solutions in their sample [16, 17], we found that an additional class fit our data better, while still having clear delineation among the classes. Macleod and colleagues (2016) identified four discrete classes, “pervasive disability”, “physical disability”, “emotional, cognitive, or interpersonal disability”, and “no/low disability” in their combined sample. More recently, in their pooled sample of older adults, Salinas-Rodríguez and colleagues (2020) found four classes of disability severity, from no to severe disability. A plausible explanation for this discrepancy may lie in the method used to derive the latent classes. As the focus of Salinas-Rodríguez et al.’s study was on severity levels, they fitted LCA models based on total WHODAS scores. Our study aim, like Macleod et al., was to identify the different types of disability faced by people with chronic conditions. To that end, responses were dichotomized to reflect item probabilities of endorsing moderate to extreme difficulty in the 12 WHODAS 2.0 items. Even so, the classes identified in this study were very similar to those found in Macleod et al.’s – Classes One, Two, Three and Five, as identified in our model, corresponded to Classes One, Three, Two and Four of Macleod et al.’s four-class model. The additional “emotional difficulty” class found in this study may be a reflection of our sample, which comprised a greater proportion of people diagnosed with depressive and anxiety disorders who in turn had a greater likelihood of being emotionally affected by their health problems as opposed to those with either a schizophrenia spectrum disorder or diabetes. Ultimately, the results of this study can be seen as an extrapolation of that found by prior studies, with the additional class adding nuance to the disability profiles captured by the WHODAS 2.0.

In the multinomial regression analysis, a significant association was found between Malay or Indian ethnicity and Class One membership, which denotes extensive difficulty. This finding is in line with those of previous studies on disability and health-related QOL – in their study on older Singaporean adults, Mahesh and colleagues [25] found higher mean WHODAS 2.0 scores in Malays and Indians compared to their Chinese counterparts. Similarly, using data from a nationwide study, Subramaniam and colleagues [26] found that those of Malay ethnicity had higher unadjusted odds of reporting some or greater levels of disability than those of Chinese ethnicity. Separately, Indian ethnicity was also found to be associated with higher odds of belonging to the physical difficulty class. Findings on the ethnicity-physical health correlation in the local population have shown that those of Indian ethnicity tended to fare worse in terms of physical health. Thumboo and colleagues (2003) found in their study that Indian adults reported lower physical health-related QOL compared to their Chinese and Malay counterparts [27]. More recently, in their analysis on gait speed, a clinical indicator of functional mobility, Shafie and colleagues (2016) found a significant association between Indian ethnicity and slower gait among the elderly in Singapore [28]. The results of our study are in alignment with those previously found, and additionally demonstrate the feasibility of the WHODAS 2.0 as a quick screening tool to capture any physical needs that should be addressed in conjunction with the treatment needs of those with chronic physical and mental health conditions.

In addition, significant associations were found between education and class memberships. Compared to a primary education, having a tertiary or university education was associated with lower odds of having extensive, social and cognitive, and emotional difficulty. This is in line with the existing literature which have established the link between disability and lower levels of education [16, 29, 30]. Education level is a known proxy of socioeconomic status [31, 32], which in turn has known associations with better access to healthcare [33] and thus better overall health. More directly, in addition to the inculcation of skills that improve help-seeking attitudes and healthy behaviors [34, 35], education improves health literacy [36, 37]. This in turn strengthens the individual’s ability, understanding and motivation towards navigating the health system to take care of their health and prevent disease [38], or to manage their disease well [39]. Hence, accounting for the education level of people entering treatment services for their chronic conditions and focusing the dissemination of health information, specifically available resources and basic self-management, on people with primary level education and below will help to mitigate the burden of disability.

Finally, we found that those who were unemployed had significantly higher odds of belonging to the extensive disability class, in line with the health-employment link that has been found in prior studies. In their systematic review on the health effects of employment, van der Noordt and colleagues [40] concluded that being employed had a protective effect especially against depression and mental health. On the other end, Norström and colleagues [41] found that unemployment contributed significantly to deterioration in individuals’ ability to carry out their usual activities, and to mood and anxiety issues. Disability can also be a useful proxy in ascertaining employability – those who experience greater disability and poorer health are more likely to become or stay unemployed, as found in Strully’s [42] study on working-age adults in the United States. This bi-directionality between employment and disability is reflected in our findings, which suggests that the WHODAS 2.0 can be a useful screener for detecting people’s employment issues in conjunction with the difficulties they may be facing in their daily lives.

The results of the study should be viewed in light of its strengths and limitations. The large sample sizes yield sufficient power for comparison of the structure and size of the latent classes across the four diagnostic sub-groups. However, as only people with specific conditions (schizophrenia, depression, anxiety and diabetes) were included in this analysis, the results are not generalizable to people with other chronic conditions. Moreover, due to the cross-sectionality of the analyses, the latent classes can only be viewed as a snapshot of one timepoint, and it is unknown whether the class structure will remain the same across time, or if it will fluctuate. Nonetheless, the results of this study demonstrate the stability of the WHODAS 2.0 in demarcating the different disability patterns faced by people with chronic conditions. This can be determined quickly and efficiently by the WHODAS 2.0 without the need for expert interviews or lengthy clinical consultations. In turn, health policy formulations can be facilitated to balance the larger scale of community health interventions for people with different health conditions while taking into account their specific disability patterns.

## Conclusion

In this study, latent disability classes were first derived from responses on the 12-item WHODAS 2.0, and potential structural and size differences between people with different conditions were investigated. The results revealed the similarity in disability patterns between people with a chronic physical or mental health condition, specific to the four conditions examined. Additionally, statistically significant sociodemographic correlates of the disability patterns were found, specifically education and ethnicity. Overall, our results demonstrate the feasibility of using the 12-item WHODAS 2.0 to quickly and efficiently screen for patterns of disability as experienced by people with chronic conditions. From this initial screening, clinicians and healthcare professionals can then promptly address their patients’ immediate functioning concerns in conjunction with chronic condition-specific symptoms that require longer-term treatment and follow-up. Ultimately, having a tool such as the WHODAS 2.0 to capture and facilitate timely follow-ups on people with disability will be essential in tailoring treatment programs that will fit with the patient’s specific needs and yield improved functional and clinical outcomes.

## Data Availability

The data is available from the authors on reasonable request.
